# Methylation detection in percutaneous lung biopsy preservation fluid: a new strategy to improve lung cancer diagnosis

**DOI:** 10.3389/fonc.2025.1611244

**Published:** 2025-07-24

**Authors:** Guang Yang, Kai Feng, Qing Tian, Yanhong Yang, Wenhao Wang, Huining Liu

**Affiliations:** ^1^ Department of Thoracic Surgery, The First Hospital of Hebei Medical University, Shijiazhuang, Hebei, China; ^2^ Department of Thoracic Surgery, Hebei Petrochina Central Hospital, Shijiazhuang, Hebei, China; ^3^ Department of Pathology, The First Hospital of Hebei Medical University, Shijiazhuang, Hebei, China

**Keywords:** lung cancer, percutaneous biopsy, preservation fluid, gene methylation, diagnostic efficacy

## Abstract

**Introduction:**

Lung cancer is a significant global health threat, particularly in China, where it has high incidence and mortality rates. Current diagnostic methods face challenges, especially in obtaining sufficient tissue samples for accurate diagnosis. This study aimed to develop a new strategy using percutaneous lung biopsy preservation fluid for gene methylation detection to improve diagnostic accuracy.

**Methods:**

A total of 182 patients who underwent percutaneous lung biopsy were included in the study. The preservation fluid was replaced with a formalin-free solution for sample collection. DNA was extracted from these samples for methylation detection of SHOX2 and RASSF1A genes.

**Results:**

The results showed that the DNA concentration qualification rate of the preservation fluid was 98.4%, and the internal reference Ct value qualification rate was 97.6%. New cut-off values were established (▵Ct_SHOX2_ = 6.16, ▵Ct_RASSF1A_ = 6.85). The combined methylation detection had a sensitivity of 92.8% and a specificity of 94.7%. When combined with traditional morphological pathology, the sensitivity increased to 96.0%. The methylation detection was more sensitive than traditional pathology, especially for early-stage and unclassified lung cancer patients. It also compensated for 82.6% of tissue pathology missed diagnoses. In patients with pulmonary tuberculosis, the combined methylation detection showed 75.0% sensitivity in diagnosing tuberculosis complicated with lung cancer.

**Discussion:**

In conclusion, methylation detection in percutaneous lung biopsy preservation fluid, when combined with traditional pathology, can effectively reduce missed diagnoses and is worthy of further promotion.

## Introduction

Lung cancer poses a significant challenge in the field of global public health. In China, its incidence and mortality rates remain persistently high. Data from the National Cancer Center in 2024 show that there were 1.0606 million new lung cancer cases and 733,300 deaths, ranking first among malignant tumors (1). Traditional diagnostic methods include imaging examinations, bronchoscopy, percutaneous biopsy, pathological diagnosis, and molecular testing. To obtain tissue samples for a definite diagnosis, for peripheral lung lesions that are determined by imaging to be inaccessible by bronchoscopy, percutaneous biopsy combined with tissue pathology is usually performed.

Percutaneous biopsy can obtain lesion tissues relatively accurately under the guidance of CT or ultrasound, and its pathological diagnosis result is regarded as the gold standard for the diagnosis of lung cancer (2). However, there are still some clinical pain points that need to be addressed. Firstly, patients are often fearful of invasive diagnostic methods such as percutaneous biopsy. Due to poor patient tolerance and information asymmetry between doctors and patients, clinicians tend to control the number of biopsy samplings. However, limited tissue samples are usually only sufficient for routine morphological pathological tests in the pathology department. As a result, some patients undergo biopsy but fail to obtain a definite pathological diagnosis due to atypical cell morphology, and clinicians cannot provide accurate treatment. Secondly, for the pathology department, due to the limited number of samples, it is necessary to prioritize meeting the needs of routine tests for most patients. When additional immunohistochemistry or molecular tests are required for samples with difficult diagnoses, it is often impossible due to insufficient tissue samples. This not only affects the diagnosis and treatment efficiency but also hinders the development of pathological diagnosis technology.

To improve the sensitivity of traditional morphological pathological diagnosis, detection techniques based on epigenetic biomarkers have gradually attracted attention. Gene methylation, as an epigenetic regulation mechanism, is closely related to tumor development (3). Methylation detection of relevant tumor suppressor genes in patients can help determine the malignancy of samples and assist in clinical decision-making. Research shows that gene methylation detection of bronchoalveolar lavage fluid can effectively improve diagnostic efficacy and reduce clinical misdiagnosis. The study by Zhang et al. showed that the diagnostic sensitivity and specificity of SHOX2 and RASSF1A gene methylation detection in bronchoalveolar lavage fluid were 72.8% and 87.1% respectively. However, due to the limitations of bronchoscopic sampling, the diagnostic sensitivity for peripheral lung adenocarcinoma is only 52.5% (4). In recent years, with the in-depth clinical application of tumor gene methylation detection technology, researchers have been constantly exploring how existing technologies can change our diagnosis and treatment process, making it more efficient without increasing the economic and psychological burden on patients.

This study attempts to start from both clinical needs and the diagnostic needs of the pathology department. Without changing the percutaneous puncture surgical procedure, more tissue or cell samples available for testing are provided to the pathology department. First, the commonly used formalin preservation solution after percutaneous biopsy is replaced with a cell preservation solution containing ethanol, phosphate, surfactant, and preservative to avoid the impact of formalin on the accuracy of gene detection. Then, as soon as the percutaneous biopsy specimen is sent to the pathology department, 5 mL of the preservation solution is aspirated and stored at 4°C for gene methylation detection, while the remaining samples are transferred to a new formalin preservation solution for routine pathological diagnosis. This study is the first to propose making full use of the preservation solution that would otherwise be discarded to achieve sample diversion for traditional pathological diagnosis and methylation detection, providing a new strategy to solve the problem of insufficient pathological samples. At the same time, the feasibility of methylation detection of biopsy preservation fluid and its diagnostic efficacy in combination with morphological pathology are demonstrated with data.

## Methods

### Patients and clinical specimens

A total of 182 patient samples who underwent percutaneous lung biopsy in the thoracic surgery department from January 2023 to October 2024 and their corresponding pathological diagnosis results were collected in this study. The final clinical diagnosis of the patients was used as the gold standard. Among the 182 patients, 125 were patients with different types of lung cancer, and 57 were in the control group. The use of percutaneous puncture surgery was determined based on the comprehensive judgment of patients’ imaging results and previous treatment results. The diagnosis of lung cancer was determined by the tissue pathology after surgery or biopsy tissue pathology combined with other clinical indicators of the patients. This study has been approved by the Clinical Research Ethics Committee of the First Hospital of Hebei Medical University, with the approval number S00477.

### Percutaneous lung biopsy and pathological diagnosis

According to the location of the patient’s lesion, an appropriate imaging guidance method was selected, and the optimal puncture path was obtained by taking the appropriate position. A biopsy sample of the lesion site was obtained using a puncture needle and placed in 10 mL of formalin-free preservation solution, and then sent to the pathology department for diagnosis. The main components of the preservation solution included ethanol, phosphate, surfactant, and preservative, and the preservation solution was purchased from Hologic.

After receiving the samples, the pathology department first aspirated 5 mL of the preservation solution with a pipette and transferred it to a new centrifuge tube, and recorded the patient information. The percutaneous biopsy samples were transferred to a preservation solution containing 10% formalin, fixed for 6–24 hours, and then paraffin-embedded tissues were prepared for pathological diagnosis.

### DNA extraction, bisulfite treatment, and methylation detection

The procedures for DNA extraction and bisulfite purification were consistent with previous reports (5). The kits were purchased from Beijing Jiaheng Yongtai Technology Co., Ltd. After purification, the bisulfite-converted DNA was amplified in parallel tubes by multiple methylation-specific real-time PCR (MS-PCR). MS-PCR amplified methylated SHOX2 (VIC), RASSF1A (FAM), and β-ACTB (CY5) DNA, with β-ACTB serving as an internal control for quantifying the total input DNA. PCR amplification targeting sequences modified with sodium bisulfite was detected using TaqMan probes. We used a Qubit DNA quantitator (purchased from Thermo Fisher) to perform quantitative analysis on the extracted cell-free DNA, and fixed 100 ng of the extracted DNA for bisulfite modification. The bisulfite-modified DNA was concentrated into 10 μL of elution buffer through a nylon membrane. The reaction volume was 40 μL, containing 5 μL of bisulfite-modified DNA, 250 μM dNTP, 0.8 μL of each primer (10 μM), 1.5–3 mM MgCl_2_, 20 μL of 2×Taq buffer, and 13.4 μL of double-distilled water (ddH_2_O). The reaction was carried out in a thermocycler with the following cycling parameters: 95°C for 10 min; 45 cycles of 95°C for 30 s; a specific annealing temperature of 58°C for 35 s, 72°C for 30 s; and a final extension step at 72°C for 8 min.

Primer and probe sequences were as follow, the forward primer of SHOX2 was TTGTTTTTGGGTTCGGGTT, the reverse primer of SHOX2 was CATAACGTAAACGCCTATACTCG, the probe of SHOX2 was VIC-ATCGAACAAACGAAACGAAAATTACC. The forward primer of RASSF1A was CGGGGTTCGTTTTGTGGTTTC, the reverse primer of RASSF1A was CCGATTAAATCCGTACTTCGC, the probe of RASSF1A was FAM-TCGCGTTTGTTAGCGTTTAAAGT. The forward primer of β-ACTB was AAGATA GTGTTGTGGGTGTAGGT, the reverse primer of β-ACTB was CCTACTTA ATACACACTCCAAAAC, the probe of β-ACTB was CY5-ACACCAACCTCATAACCTTATCACAC-BHQ.

Sulfite was used to modify unmethylated cytosine bases to uracil bases, which were then converted to thymine bases during PCR amplification. Methylated cytosine bases remained unchanged, enabling the differentiation between methylated and unmethylated cytosine bases. Specific primers targeting sequences after bisulfite modification were designed for PCR. PCR amplification products were detected using Taq-Man probes (85 nM). A plasmid containing the corresponding methylated gene sequence was used as the positive control, and purified water was used as the negative control. The β-actin gene served as the internal control for quantifying the total input DNA. The baseline position was adjusted (Threshold = 10000; Noise band = 0.8) to obtain the Ct value of the fluorescence signal. The Ct value of the β-actin gene signal should be between 18 and 23. When performing multi-gene combined detection, since the functions of each gene were independent of each other, if any one gene was positive, the result of the combined test was judged as positive. Relative levels of SHOX2 and RASSF1A methylation were calculated by the delta cycle threshold (ΔCt). To enhance the stability of the quantitative real-time PCR (qPCR) results and to mitigate the influence of non-specific amplification, the fluorescence signal intensity of the target gene was normalized by subtracting the corresponding signal from an endogenous housekeeping gene. This normalization step is crucial for obtaining reliable and reproducible gene expression data, as it accounts for variations in sample quality, reverse transcription efficiency, and PCR amplification efficiency. As follows: ΔCt_SHOX2_ = Ct_SHOX2_ – Ct_β-actin_; ΔCt_RASSF1A_ = Ct_RASSF1A_ – Ct_β-actin_.

### Statistical analysis

Statistical analysis was performed using the SPSS 19.0 software package (SPSS Inc., Chicago, IL). The receiver operating characteristic curve (ROC) was used to calculate the Youden index and the area under the ROC curve (AUC) to evaluate the diagnostic efficacy. Statistical significance was set at a *P* value < 0.05.

## Results

### Patient characteristics

A total of 182 patients were included in this study, among whom 125 were diagnosed with lung cancer and 57 were in the benign disease control group. As shown in **Table 1**, the age range of patients in the lung cancer group was 30–84 years, with an average age of 62 years, a median age of 65 years, and the proportion of patients in the 50 - 69-year-old age group was 68.8%, which was significantly higher than that in other age groups (P = 0.036). In the benign control group, the age range was 31–82 years, with an average age of 58 years, a median age of 57 years, and the 50 - 69-year-old age group had the highest proportion, accounting for 71.9%. After analyzing gender, it was found that the proportion of males in the lung cancer group was 67.2%, and that in the control group was 75.4%, both significantly higher than that of female patients (P < 0.001). Among the 125 lung cancer patients, 61.6% were adenocarcinoma patients, 14.4% were squamous cell carcinoma patients, 5.6% were small cell lung cancer patients, and 18.4% were unclassified lung cancer patients. The proportion of adenocarcinoma patients was significantly higher than that of other pathological types (P < 0.001). Among the benign control patients, 52.6% were tuberculosis patients and 47.4% were pneumonia patients.

**Table 1:** Patient demographic and clinical features.

Category	Lung cancer		Benign disease	
	n	%	n	%
Total	125		57	
Age				
30-49	13	10.4%	10	17.5%
50-69	86	68.8%	41	71.9%
≥70	26	20.8%	6	10.5%
Age range	30-84		31-82	
Average age	62		58	
Median age	65		57	
Sex				
Male	84	67.2%	43	75.4%
Female	41	32.8%	14	24.6%
Histology subtype				
Lung Cancer				
LUAC	77	61.6%		
LUSC	18	14.4%		
SCLC	7	5.6%		
Unclassified Lung Cancer	23	18.4%		
Benign Disease				
Pulmonary tuberculosis			30	52.6%
Pneumonia			27	47.4%

### Experimental quality control of methylation detection

**Figure 1:**
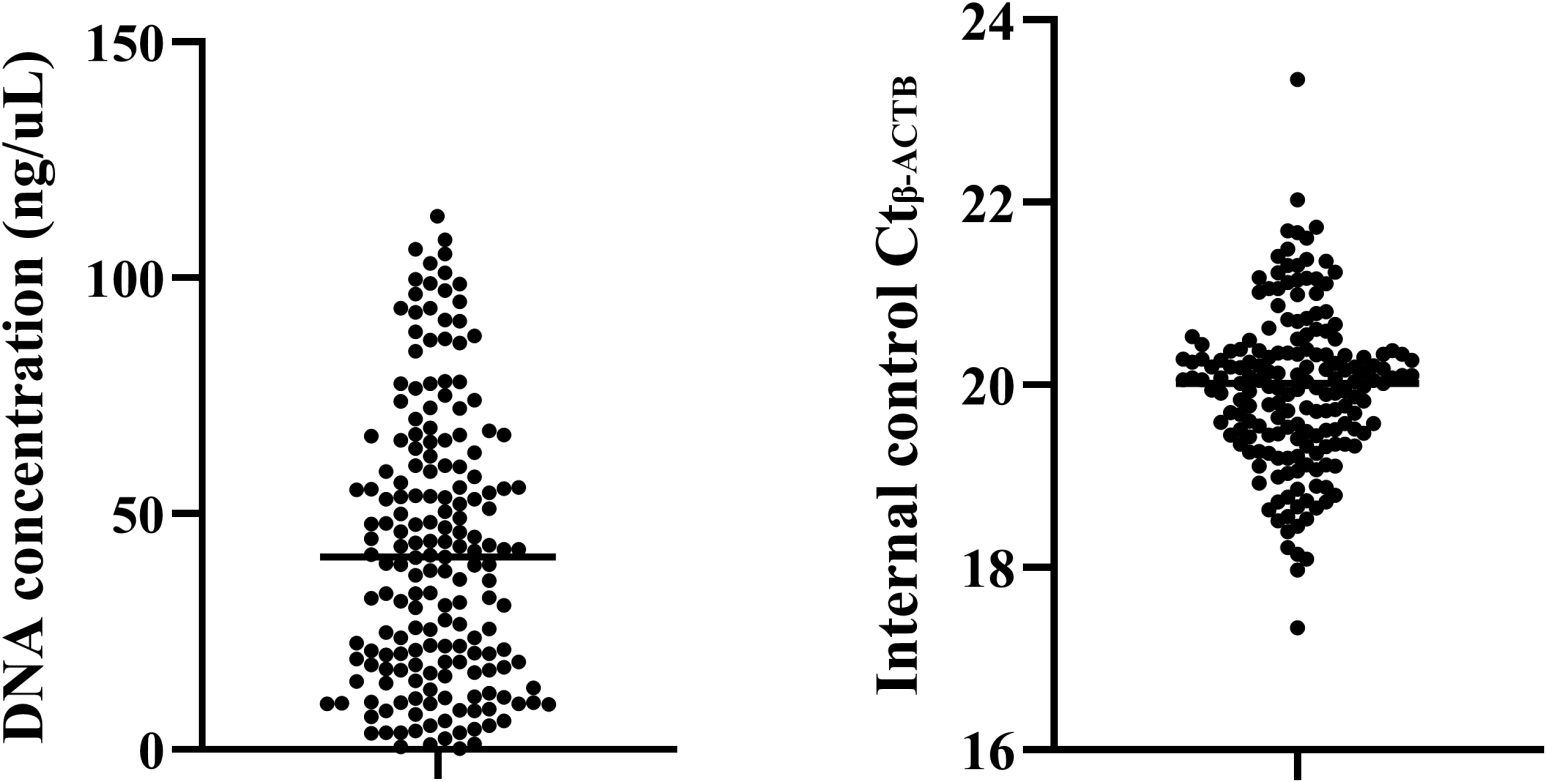
Quality control of methylation detection in percutaneous biopsy sample preservation fluid.

This study is the first to attempt DNA methylation detection using the preservation fluid of percutaneous lung biopsy. To ensure the accuracy and effectiveness of the experiment, the DNA concentration extracted from the preservation fluid was first analyzed. The results showed (**Figure 1**) that the DNA concentrations extracted from the preservation fluid of 2 patients were relatively low, 0.206 ng/μL and 0.62 ng/μL respectively, while the DNA concentrations of the remaining samples were all greater than 1 ng/μL. The DNA concentration range was 0.206–113 ng/μL, with an average concentration of 42.5 ng/μL. A DNA concentration greater than 1 ng/μL was considered qualified. The DNA concentration qualification rate was 98.4% (123/125) in the lung cancer group and 100% (57/57) in the benign disease group. In addition, an internal reference gene was set for methylation detection to monitor the PCR process and amplification efficiency. When the Ct_β-actin_ was between 18 and 23, it indicated that the amplification process was stable, and the detection results were less affected by non-specific amplification, making the results more reliable. In this study, the Ct β-actin of all samples was between 17.34 and 23.35, with a qualification rate of 97.6%.

### Setting of cut-off values for methylation detection of percutaneous biopsy sample preservation fluid

The SHOX2 and RASSF1A gene methylation combined detection kit used in this study has been approved by the National Medical Products Administration (NMPA) of China for the identification of the malignancy of exfoliated cells in bronchoalveolar lavage fluid as an *in vitro* diagnostic reagent. However, there is currently no research proving that this reagent can be used for the diagnosis of percutaneous biopsy samples and their preservation fluid. Therefore, this study needed to re-set the Cut-off values to adapt to the new sample type.

**Figure 2:**
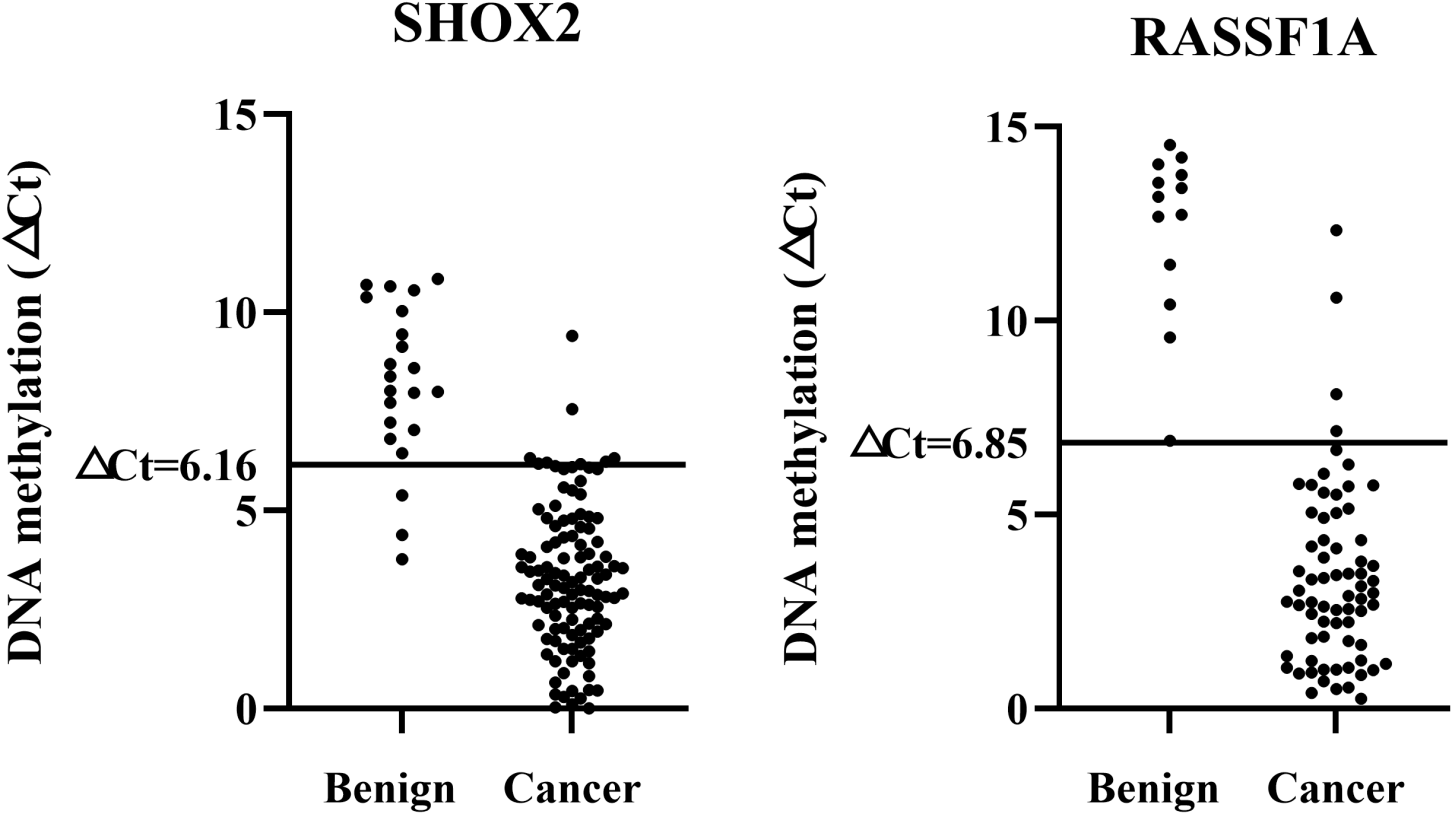
Optimal cut-off values of SHOX2 and RASSF1A.

All samples were divided into a training set and a validation set. ROC analysis was performed on the training set data, and the Cut-off values for SHOX2 and RASSF1A were determined based on the Youden index. The Cut-off value corresponding to the maximum Youden index for SHOX2 was 6.16. At this point, the detection sensitivity of SHOX2 was 91.9%, and the specificity was 96.4%. The Cut-off value corresponding to the maximum Youden index for RASSF1A was 6.85. At this point, the detection sensitivity of RASSF1A was 51.6%, and the specificity was 100%. To further evaluate the accuracy, the set Cut-off values were substituted into the validation set for verification. Among them, the sensitivity of SHOX2 was 81.0%, and the specificity was 93.1%. The sensitivity of RASSF1A was 52.4%, and the specificity was 100%. There was no statistical difference in sensitivity and specificity between the training set and the validation set (P > 0.05). Therefore, it is considered that ▵Ct_SHOX2_ = 6.16 and ▵Ct_RASSF1A_ = 6.85 are ideal Cut-off values and can be used for subsequent studies (**Figure 2**).

### Comparison of detection sensitivities of different methods for percutaneous lung biopsy samples and their preservation fluid

As shown in **Table 2**, the overall positive rate of SHOX2 was 87.2%. The detection sensitivities of the percutaneous biopsy preservation fluid for patients with adenocarcinoma, squamous cell carcinoma, small cell lung cancer, and unclassified lung cancer were 85.7%, 100%, 100%, and 73.9%, respectively. The overall positive rate of RASSF1A was 52%, and the detection sensitivities of the percutaneous biopsy preservation fluid for adenocarcinoma, squamous cell carcinoma, small cell lung cancer, and unclassified lung cancer were 57.1%, 27.8%, 100%, and 39.1% respectively. When the two methylation indicators were combined for detection, the diagnostic sensitivity for adenocarcinoma increased from 85.7% to 94.8%, and the diagnostic sensitivity of the two-index combined detection for adenocarcinoma was significantly higher than that of single-index detection (P_SHOX2_ < 0.01, P_RASSF1A_ < 0.001).

**Table 2:** Comparison of sensitivities of gene methylation detection and tissue pathology diagnosis in different types of patients.

Histology subtype	Total	SHOX2		RASSF1A		SHOX2+RASSF1A		Tissue pathology diagnosis	
		n	Sensitivity	n	Sensitivity	n	Sensitivity	n	Sensitivity
Lung Cancer									
LUAC	77	66	85.7%	44	57.1%	73	94.8%	69	89.6%
LUSC	18	18	100.0%	5	27.8%	18	100.0%	16	88.9%
SCLC	7	7	100.0%	7	100.0%	7	100.0%	7	100.0%
Unclassified Lung Cancer	23	17	73.9%	9	39.1%	18	78.3%	10	43.5%
Total	125	108	86.4%	65	52.0%	116	92.8%	102	81.6%
Benign Disease									
Pulmonary tuberculosis	30	2	6.7%	0	0.0%	2	6.7%	0	0.0%
Pneumonia	27	1	3.7%	0	0.0%	1	3.7%	0	0.0%
Total	57	3	5.3%	0	0.0%	3	5.3%	0	0.0%

In addition, the overall sensitivity of traditional morphological pathological diagnosis of percutaneous biopsy samples was 81.6%, which was significantly lower than the 92.8% of the combined gene methylation detection of the preservation fluid (P < 0.05). For patients with unclassified lung cancer, the diagnostic sensitivity of traditional morphological pathology was only 43.5%, which was significantly lower than that of the methylation combined detection (P < 0.01). In the control group, 2 tuberculosis patients had positive gene methylation results, with a specificity of 93.3%. The specificity of the gene methylation combined detection for pneumonia patients was 96.3%, and the overall specificity was 94.7%.

### ROC curves of different diagnostic methods

**Figure 3:**
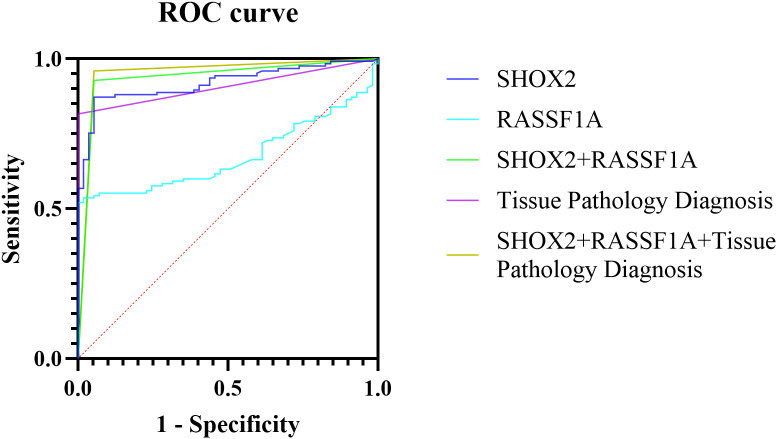
ROC curves of different methods for diagnosing percutaneous biopsy tissues and their preservation fluids.

ROC curves were drawn for the combined methylation detection results of the preservation fluid and the tissue pathology of percutaneous biopsy samples, and the AUC values were calculated (**Table 3**, **Figure 3**). The results showed that the AUC value of SHOX2 methylation detection in the preservation fluid was 0.9200 (95% CI: 0.8805 - 0.9595), with a sensitivity of 86.4% and a specificity of 94.7%. The AUC value of RASSF1A methylation detection in the preservation fluid was 0.6756 (95% CI: 0.6002 - 0.7510), with a sensitivity of 52.0% and a specificity of 100.0%. The AUC value of the combined detection of SHOX2 and RASSF1A was 0.9377 (95% CI: 0.8950 - 0.9803), which was higher than that of single-index detection. The combined diagnosis of gene methylation in the preservation fluid and tissue pathology could further increase the AUC value to 0.9537 (95% CI: 0.9147 - 0.9927), with a comprehensive sensitivity of 96.0%, a specificity of 94.7%, and positive predictive value (PPV) and negative predictive value (NPV) reaching 98.7% and 90.6%, respectively.

**Table 3:** AUC values of gene methylation detection and tissue pathology diagnosis.

Diagnostic method	AUC					
	Value	95% CI	Sensitivity	Specificity	PPV	NPV
SHOX2	0.9200	0.8805 - 0.9595	86.4%	94.7%	96.2%	89.2%
RASSF1A	0.6756	0.6002 - 0.7510	52.0%	100.0%	100.0%	70.4%
SHOX2+RASSF1A	0.9377	0.8950 - 0.9803	92.8%	94.7%	98.1%	88.9%
Tissue Pathology Diagnosis	0.9080	0.8652 - 0.9508	81.6%	100.0%	100.0%	71.3%
SHOX2+RASSF1A+ Tissue Pathology Diagnosis	0.9537	0.9147 - 0.9927	96.0%	94.7%	98.7%	90.6%

### Diagnostic sensitivities of the combined methylation detection in preservation fluid and tissue pathology diagnosis of percutaneous biopsy for patients at different stages

Among the 125 patients enrolled in this study, 103 patients had available clinical stage information. Specifically, 22 patients were in stage I, 18 in stage II, 21 in stage III, and 42 in stage IV (**Table 4**). The sensitivity of the combined gene methylation detection in the preservation fluid increased with the advancement of the patient’s stage. The sensitivities were 81.8% in stage I, 88.9% in stage II, 100% in stage III, and 97.6% in stage IV. The sensitivity of the tissue pathology diagnosis of percutaneous biopsy was lower than that of the combined gene methylation detection at each stage, with values of 72.7% in stage I, 55.6% in stage II, 81.0% in stage III, and 95.2% in stage IV. It is worth noting that tissue pathology diagnosis might issue gray - zone reports at all stages. Moreover, the earlier the stage, the higher the proportion of gray - zone reports. The proportion in stage I was 13.6%. Additionally, the missed diagnosis rates of tissue pathology for stage II and stage III patients were higher than those of the combined gene methylation detection (33.3% vs. 11.1% in stage II, 9.5% vs. 0% in stage III).

**Table 4:** Positive rates of gene methylation detection and tissue pathology diagnosis at different clinical stages.

Pathological staging	Total	Methylation			Tissue pathology diagnosis		
		SHOX2 positive	RASSF1A positive	SHOX2+RASSF1A positive	Positive	Atypical hyperplasia	Negative
		n(%)	n(%)	n(%)	n(%)	n(%)	n(%)
Stage I	22	15 (68.2%)	8 (36.4%)	18 (81.8%)	16 (72.7%)	3 (13.6%)	3 (13.6%)
Stage II	18	15 (83.3%)	6 (33.3%)	16 (88.9%)	10 (55.6%)	2 (11.1%)	6 (33.3%)
Stage III	21	20 (95.2%)	12 (57.1%)	21 (100%)	17 (81.0%)	2 (9.5%)	2 (9.5%)
Stage IV	42	39 (92.9%)	26 (61.9%)	41 (97.6%)	40 (95.2%)	1 (2.4%)	1 (2.4%)

### The combined methylation detection in preservation fluid can serve as a supplementary diagnosis to tissue pathology to reduce clinical missed diagnoses

**Figure 4:**
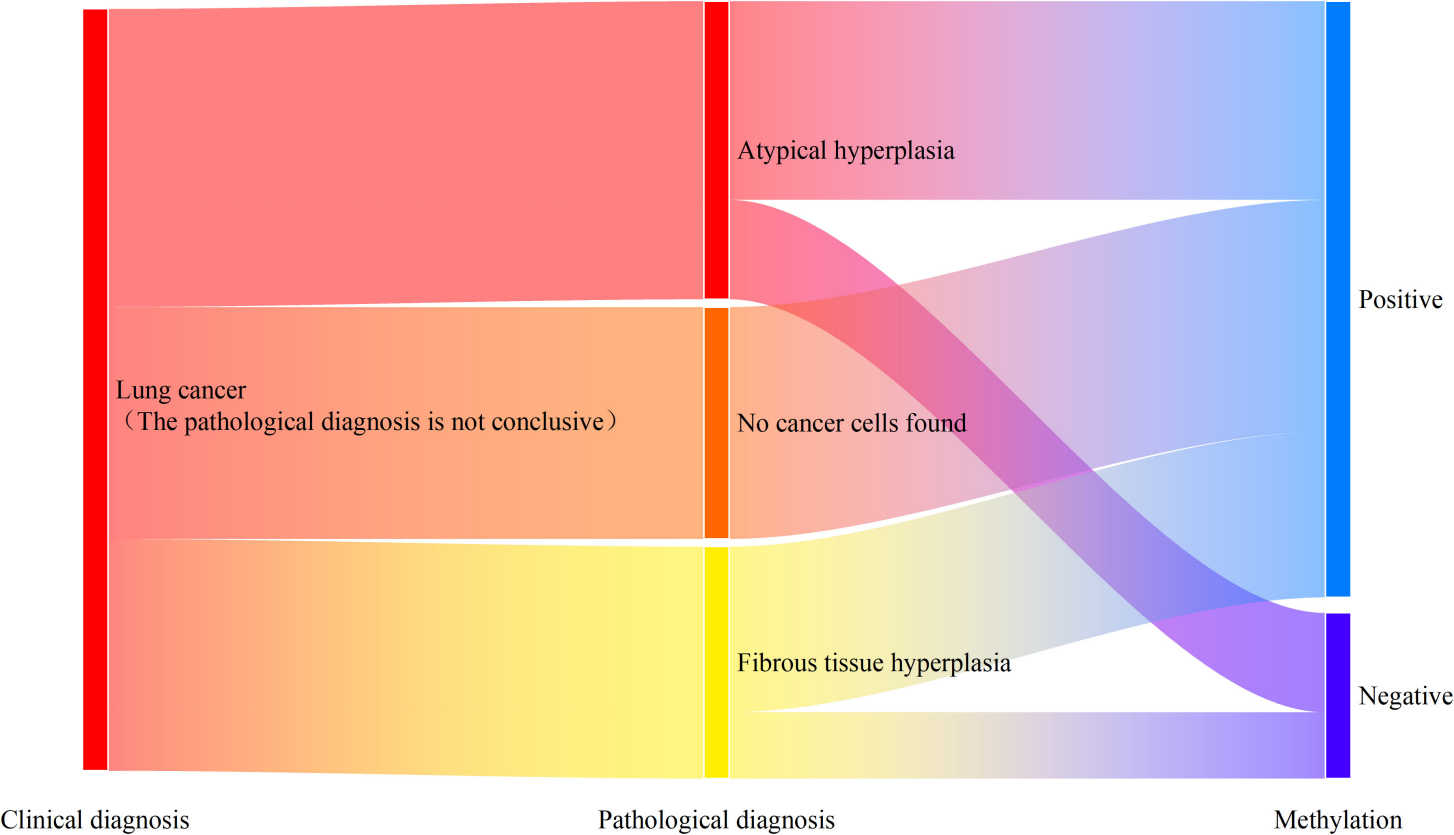
Methylation detection results of patients with missed diagnosis by percutaneous tissue pathology.

Among the patients diagnosed with lung cancer, there were 23 patients whose percutaneous biopsy tissue pathology did not report cancer cells. Among them, 18 patients (78.3%) had positive methylation detection results. As shown in **Figure 4**, among the 9 patients reported with atypical hyperplasia, 6 patients (66.7%) had positive results in the combined methylation detection of the preservation fluid. All 7 patients reported with no cancer cells detected had positive results in the combined methylation detection of the preservation fluid. Among the 7 patients reported with fibrous tissue hyperplasia, 5 patients (71.4%) had positive results in the combined methylation detection of the preservation fluid.

**Figure 5:**
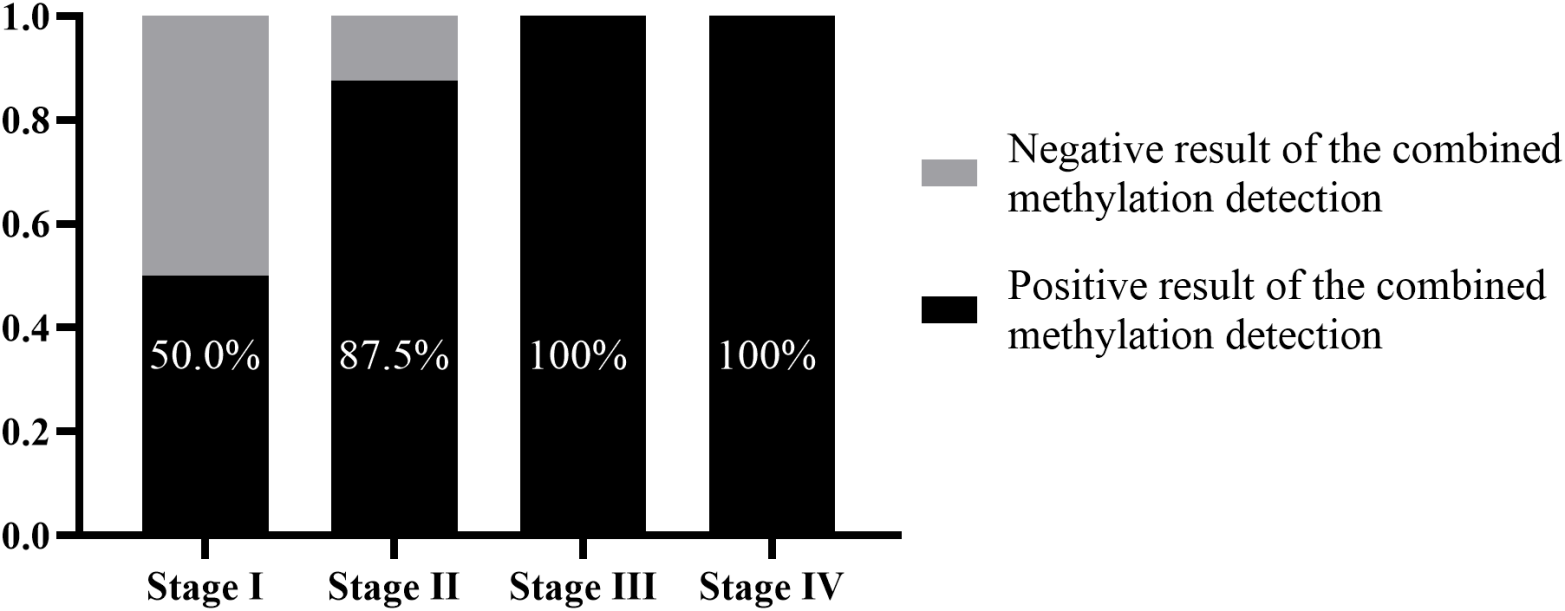
Supplementary diagnostic role of methylation detection in percutaneous biopsy preservation fluid for tissue pathology at different clinical stages.

In addition, among the 23 patients with missed diagnoses by tissue pathology, there were 6, 8, 4, and 2 patients in stages I – IV, respectively. The supplementary diagnostic effect of the combined methylation detection in the preservation fluid for stage I patients was 50.0% (3/6). For stage II patients with missed diagnoses, 87.5% (7/8) of the missed diagnoses could be compensated through the combined methylation detection. For stage III and stage IV patients, by combining tissue pathology with the combined methylation detection, clinical missed diagnoses could be completely avoided (**Figure 5**).

### Diagnostic efficacy of the combined methylation detection in patients with pulmonary tuberculosis and pulmonary tuberculosis complicated with lung cancer

Among the 182 patients enrolled in this study, 38 patients were diagnosed with pulmonary tuberculosis, and 8 of them were diagnosed with pulmonary tuberculosis complicated with lung cancer. Among the patients clinically diagnosed with pulmonary tuberculosis (30 patients, excluding those with pulmonary tuberculosis complicated with lung cancer), 20 patients (66.7%) had granulomatous lesions reported by tissue pathology, 4 patients (13.3%) had inflammatory cell infiltration, 3 patients (10.0%) had fibrous tissue hyperplasia, and 3 patients (10.0%) had necrotic tissue. Among the 20 patients with granulomatous tuberculosis, the combined gene methylation detection found 1 false - positive case, with a specificity of 95.0%. Judging from the △Ct values of the detection results, the △Ct values of the false - positive case was △Ct_SHOX2_ = 5.38. These value was close to the Cut - off values (△Ct_SHOX2 Cut-off_ = 6.16) and belonged to weak - positive results.

**Figure 6:**
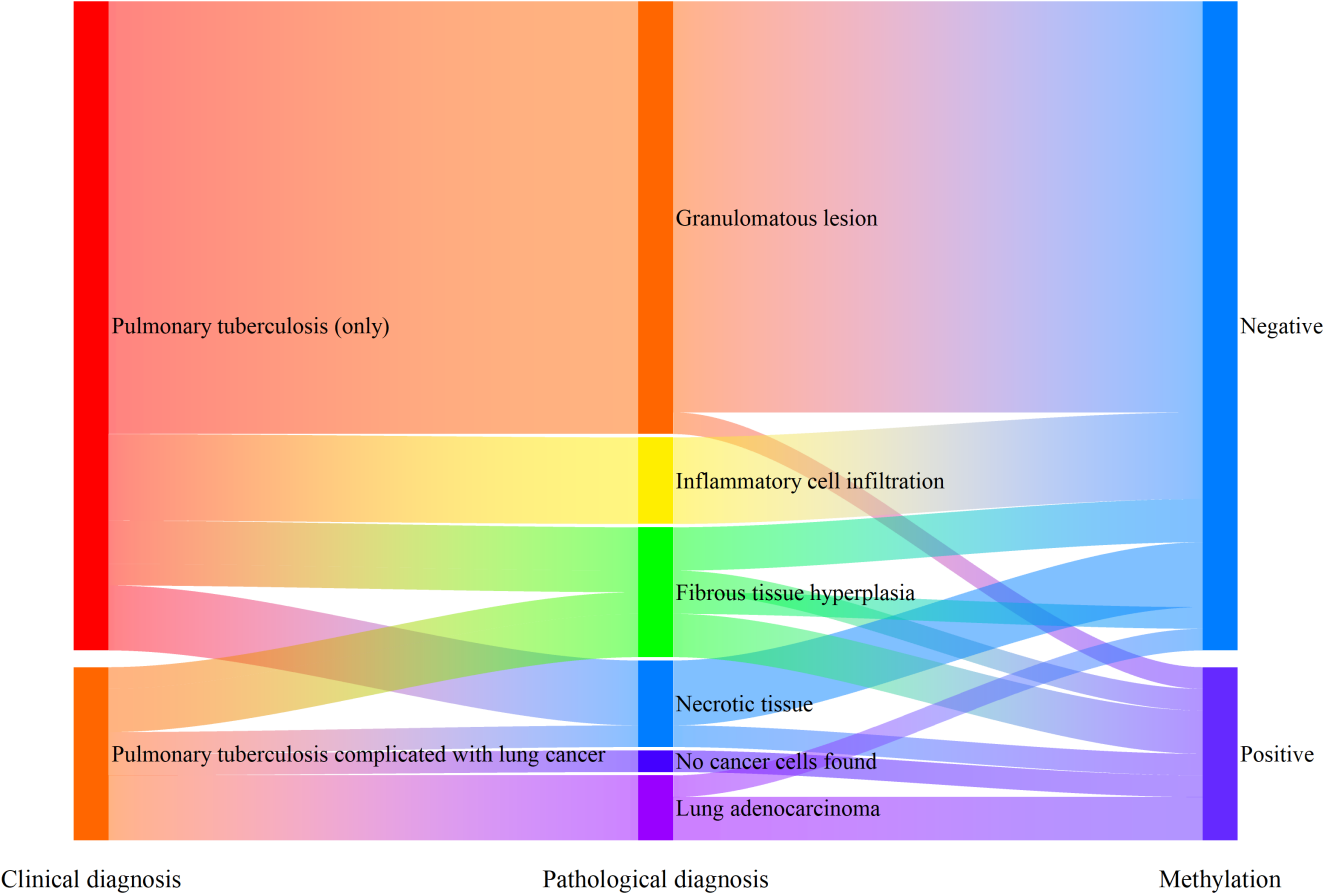
Supplementary diagnostic role of methylation detection in percutaneous biopsy preservation fluid for patients with pulmonary tuberculosis complicated with lung cancer.

For the 8 patients clinically diagnosed with pulmonary tuberculosis complicated with lung cancer, only 3 patients had lung adenocarcinoma cells detected by tissue pathology (including 2 stage IV patients). The other 5 patients included 2 stage I patients and 3 stage II patients, and tissue pathology missed the diagnosis for all of them. The diagnostic sensitivity of the combined gene methylation detection for patients with pulmonary tuberculosis complicated with lung cancer could reach 75.0%, effectively compensating for the shortcomings of tissue pathology diagnosis (**Figure 6**).

## Discussion

Lung cancer is the leading cancer threatening human health. With the rapid development of technologies such as low - dose spiral CT, EBUS-TBNA, rapid on-site evaluation (ROSE), and gene methylation detection, the early diagnosis technology for lung cancer has significantly improved. The initial diagnosis of peripheral lung lesions mainly relies on imaging and the experience of clinicians. Percutaneous lung biopsy and tissue pathology diagnosis remain the gold standard for diagnosing peripheral lung lesions. However, due to various factors such as inappropriate puncture sites, insufficient puncture sampling volume, non - standard pathological slide preparation in primary hospitals, tumor heterogeneity, and lack of experience of pathologists, there is still a certain proportion of missed diagnoses in clinical practice (6–8).

As an invasive diagnostic method, adding molecular pathological diagnosis with higher sensitivity and more objective detection results to traditional pathological diagnosis in percutaneous biopsy helps reduce the probability of diagnostic failure, and there have been successful precedents in various solid tumors. Existing studies have shown that simultaneous morphological pathological diagnosis and BRAF V600E gene mutation detection in thyroid fine-needle aspiration biopsy samples can reduce the missed diagnosis rate of suspicious papillary thyroid carcinoma by 52% (9–11). In addition, studies have shown that BRCA gene mutation detection in breast percutaneous biopsy samples can assess the risk of breast cancer and help determine whether prophylactic mastectomy is needed (12). In the field of lung cancer diagnosis and treatment, genetic testing of lung percutaneous biopsy samples is mainly used to evaluate the value of targeted drug use, such as detecting gene mutations such as EGFR and K-RAS. Walter et al.’s study showed that methylation detection of L1RE1, RARB, and RASSF1 genes in lung cancer tissue samples can effectively distinguish between benign and malignant tumors with a specificity of 91% (13). However, the traditional diagnostic methods adopted by the vast majority of medical institutions have not solved the problem in actual clinical practice that the volume of percutaneous puncture biopsy specimens is too small to simultaneously meet the comprehensive diagnostic needs of morphological and molecular pathology.

Regarding the problem of difficult gene detection in small biopsy samples, researchers have carried out explorations. The study by Jiang et al. developed a protocol for collecting cells from the fixative medium of transbronchial lung biopsy samples and using them for gene mutation detection (14). This protocol enabled gene detection in 75.7% of patients with insufficient sample volume, effectively improving the mutation detection rate. The study extracted genomic DNA from cell pellets in the fixative medium and obtained high-quality cellular genomic DNA, significantly reducing the demand for microdissection in pathology departments and improving the work efficiency of pathology departments. Pagni et al. also proposed a liquid shaking method (15). By gently shaking the normal saline preservation solution of biopsy samples, high-quality DNA and living cells were obtained for molecular detection. This method can obtain an average of 146 ng/μL of DNA, which is significantly higher than that extracted from FFPE samples, providing a technical basis for supplementary gene detection of hematological diseases and solid tumors.

The above studies have fully demonstrated the feasibility of using exfoliated cells in the preservation fluid of biopsy samples for gene detection. In our study, a quantitative analysis of cell-free DNA in the preservation fluid found that the samples of 98.4% in the lung cancer group and 100% in the benign disease group could extract DNA above 1 ng/μL. Under the premise that the pathology department of our hospital uses an automated cell-free DNA extractor and the DNA elution volume is 70 μL, it can ensure that low-concentration DNA samples also obtain an internal reference Ct value of 20-24. When genomic DNA extracted from exfoliated cells is usually used for methylation detection, the internal reference Ct value is 18-23. The difference between the two is not obvious, but the automated equipment significantly reduces the workload of pathologists. In future studies, we will consider conducting a comparative study on the diagnostic efficacy of exfoliated cells and cell-free DNA to evaluate the impact of different DNA extraction protocols on the detection results.

Why, as the highest incidence cancer, there is still no widely recognized gene detection project in China’s clinical practice? We believe this is related to the fixed work habits of clinicians and pathologists over the years. Clinicians tend to minimize the sample volume of percutaneous biopsies to avoid increasing the burden on patients. Facing a small number of samples, pathologists can only prioritize the most routine tests. This study explores the use of gene methylation detection in biopsy preservation fluid, which does not require clinicians to increase the sample volume of percutaneous biopsies or have complicated communication with patients, and hardly changes the original work mode. At the same time, the pathology department can convert the originally discarded preservation fluid into effective samples that can enhance diagnostic efficacy, making additional molecular diagnosis possible.

This study is the first to use gene methylation detection in biopsy preservation fluid. Therefore, the quality control of the experiment was verified, and the Cut - off values were reset. In terms of experimental quality control, 98.4% of the percutaneous biopsy preservation fluid could extract sufficient DNA for methylation detection. Moreover, 97.6% of the internal reference Ct values were between 18 and 23, indicating reliable results. The detection reagent used in this project is a marketed product approved by the National Medical Products Administration (NMPA) of China, but its approved indication is the auxiliary diagnosis of bronchoalveolar lavage fluid. When used for methylation detection of puncture preservation fluid, we redrew the ROC curve and calculated the Youden index of SHOX2 and RASSF1A. It was found that when △Ct_SHOX2_ = 6.16 and △Ct_RASSF1A_ = 6.85, the detection sensitivity and specificity reached the optimal level.

In this study, the sensitivity of the tissue pathology diagnosis of percutaneous biopsy was 81.6%. When combined with imaging, tumor markers, and other indicators for comprehensive diagnosis, it can basically meet the needs of clinicians. After incorporating the methylation detection of percutaneous biopsy preservation fluid, the diagnostic sensitivity further increased to 96.0% (AUC = 0.9537), and the missed diagnosis situation was better improved. It should be emphasized that as a regional teaching hospital, our hospital has a relatively high level of morphological pathology diagnosis. As a result, based on the experimental data, the improvement in the diagnostic efficacy of the methylation detection of percutaneous biopsy preservation fluid for the tissue pathology diagnosis of percutaneous biopsy samples is not very significant. However, China is a developing country with a vast territory and a large population. The levels of morphological pathology diagnosis in a large number of primary hospitals vary greatly, often resulting in patients being unable to determine the nature of the lesions even after undergoing percutaneous lung biopsies. The methylation detection of percutaneous biopsy preservation fluid, as a more objective detection method, not only improves the clinical accessibility of molecular detection of percutaneous biopsy samples but also, to a certain extent, makes up for the differences in diagnostic levels among hospitals of different grades, helping to achieve accurate diagnosis before patient treatment.

For early - stage patients, tissue pathology diagnosis is more difficult and is more easily affected by factors such as sampling sites, cell morphology, and doctor experience, with a relatively higher missed diagnosis rate. In this study, the missed diagnosis rates of stage I and stage II patients were 27.3% and 44.4% respectively. Among the 8 patients with tuberculosis complicated with lung cancer, 5 stage I and stage II patients had missed diagnoses. As an auxiliary diagnostic method that has developed rapidly in recent years, gene methylation detection has a significant supplementary diagnostic effect on early - stage patients. The study by Zhang et al. showed that the methylation detection sensitivity of bronchoalveolar lavage fluid for stage I patients was 85.7%, and for stage II patients, it was 80.0%, both higher than the 46.4% and 56.7% of morphological pathology diagnosis (16). The study by Ren et al. showed similar results, with diagnostic sensitivities of 70.2% and 61.5% for stage I and stage II patients respectively (17). With the widespread application of low - dose spiral CT in China, the positive rate of early - stage lung cancer screening has significantly increased in recent years. More and more patients with malignant pulmonary nodules have been screened out in stage I or even at the in - situ carcinoma stage. However, the development of traditional morphological pathology has not kept pace with the level of early - stage lung cancer screening. There is an urgent need for more diversified diagnostic methods in clinical practice to determine the malignancy of patients’ lesions and provide more accurate treatment in a timely manner. The methylation detection of percutaneous biopsy preservation fluid can provide more patient samples for testing in the pathology department without increasing the workload of clinicians and the burden on patients.

Tissue pathology diagnosis is the gold standard for the diagnosis of lung cancer. When tissue pathology diagnosis does not detect cancer cells or issues a gray - zone report, it is difficult for clinicians to make accurate judgments based solely on patients’ symptoms and imaging examinations. This study found that when gene methylation detection was performed on all percutaneous biopsy samples, 82.6% of the missed diagnoses by tissue pathology could be compensated. The supplementary diagnostic effects on stage I and stage II patients were 50.0% and 87.5% respectively.

It is also worth noting that in this study, we found that the combined methylation detection of percutaneous biopsy preservation fluid in patients with granulomatous tuberculosis may yield positive results. In another ongoing study we participated in, 9% of the pleural effusion samples from tuberculosis patients showed positive methylation of PTGER4 and/or SHOX2 genes. In addition, there is an ongoing study showing that when gene methylation detection was added to the bronchoalveolar lavage fluid during bronchoscopy for tuberculosis patients, 6.9% of the tuberculosis patients were diagnosed with concurrent malignant tumors. Before the methylation detection, their malignant tumors were missed. Some published studies also show that the probability of secondary lung cancer in tuberculosis patients, especially those with active and refractory pulmonary tuberculosis, increases significantly (18, 19). In clinical practice, tuberculosis patients, especially those with granulomatous lesions, should actively screen for the possibility of lung malignancies to avoid delaying treatment opportunities.

The limitations of this study are that it is a single-center study and the sample size is relatively small, which makes it impossible to determine unique Cut - off values for samples of different pathological types. In future research, we will consider including more patient samples to enhance the rigor of data statistics.

In conclusion, in this study, through the methylation detection of SHOX2 and RASSF1A genes in the preservation fluid of percutaneous lung biopsy samples, a diagnostic sensitivity of 92.8% and a diagnostic specificity of 94.7% were achieved. When the methylation detection was combined with traditional morphological pathology diagnosis, the sensitivity further increased to 96.0%, while the specificity remained at 94.7%. Through the comprehensive diagnosis of traditional pathology and gene methylation detection, the number of pathological gray - zone reports were effectively reduced, thereby reducing the occurrence of clinical missed diagnoses and enabling patients to receive more accurate treatment in a timely manner. This method is worthy of further promotion and application.

## Data Availability

The raw data supporting the conclusions of this article will be made available by the authors, without undue reservation.
